# Nipple-sparing Mastectomy with Immediate Implant-based Reconstruction for Patients with Pure Ductal Carcinoma in Situ

**DOI:** 10.1055/s-0042-1742315

**Published:** 2022-05-27

**Authors:** Antônio Luiz Frasson, Ana Beatriz Falcone, Isabela Miranda, Alessandra Borba Anton de Souza, Betina Vollbrecht, Fernanda Barbosa, Mônica Adriana Rodriguez Martinez Frasson, Martina Lichtenfels

**Affiliations:** 1 Hospital Israelita Albert Einstein, São Paulo, SP, Brazil; 2 Mastology Service, Hospital São Lucas, Pontifícia Universidade Católica do Rio Grande do Sul, Porto Alegre, RS, Brazil

**Keywords:** breast cancer, intraductal noninfiltrating carcinoma, subcutaneous mastectomy, neoplasias da mama, carcinoma ductal de mama, mastectomia subcutânea

## Abstract

**Objective** The presence of an extensive intraductal component is associated to an increasing risk of relapse in the nipple-areola complex. The aim of the present study was to evaluate the outcomes of patients diagnosed with ductal carcinoma in situ (DCIS) who underwent nipple-sparing mastectomy (NSM) with immediate breast reconstruction using silicone implants.

**Methods** We retrospectively analyzed the postoperative complications and oncological safety of 67 breast cancer patients diagnosed with pure DCIS who underwent NSM with immediate breast reconstruction using silicone implants between 2004 and 2018.

**Results** Among the 127 NSM procedures performed, 2 hematomas (1.5%) and 1 partial nipple necrosis (0.7%) were observed. After a mean follow-up of 60 months, the local recurrence rate was of 8.9%, the disease-free survival rate was of 90%, and 1 of the patients died.

**Conclusion** Despite the local recurrence rate, we showed that NSM with immediate breast reconstruction using silicone implants is a feasible surgical approach, with a low rate of complications and high survival rates for patients with a diagnosis of pure DCIS when breast-conserving surgery is not an option.

## Resumo

**Objetivo** A presença de componente intraductal extenso é associada ao risco aumentado de recorrência no complexo aréolo-mamilar. O objetivo deste estudo foi avaliar os resultados de pacientes diagnosticados com carcinoma ductal *in situ* (CDIS) submetidas a adenomastectomia ( *nipple-sparing mastectomy* , NSM, em inglês) com reconstrução mamária imediata utilizando prótese de silicone.

**Métodos** Restrospectivamente, foram analisadas as complicações pós-operatórias e a segurança oncológica de 67 pacientes com câncer de mama diagnosticadas com CDIS puro, e submetidas a NSM com reconstrução mamária imediata utilizando prótese de silicone, entre 2004 e 2018.

**Resultados** Entre os 127 procedimentos realizados, 2 hematomas (1,5%) e 1 necrose parcial de mamilo (0,7%) foram observados. Após um período médio de 60 meses de seguimento, a taxa de recorrência local foi de 8,9%, a sobrevida livre de doença, de 90%, e apenas 1 paciente foi a óbito.

**Conclusão** Apesar da taxa de recorrência local, demostrou-se que NSM com reconstrução mamária imediata com prótese de silicone é um procedimento viável, com baixa taxa de complicação e alta sobrevida para pacientes com diagnóstico de CDIS puro quando a cirurgia conservadora da mama não é uma opção.

## Introduction

Nipple-sparing mastectomy (NSM) has been successfully performed in the treatment of breast cancer, with excellent results. 1 2 3 4 The indications for NSM include invasive breast cancer and ductal carcinoma in situ (DCIS) for patients in whom breast-conserving surgery (BCS) may not be performed. 5 Some contraindications to BCS include larger tumor sizes, multifocal and multicentric tumors, contraindications to radiotherapy (RT), a potentially-poor cosmetic outcome due to the tumor/breast relationship, and patient choice. 5 6 Currently, some authors 7 suggest expanding NSM indications to include patients with large tumors who have undergone neoadjuvant chemotherapy, and patients with local recurrence after BCS followed by RT. The presence of an extensive intraductal component is strongly associated to an increasing risk of relapse in the nipple-areola complex. 8 Previous studies 3 9 10 have reported low rates of recurrence in the nipple-areola complex, ranging from 1.4% to 3.2%, in patients diagnosed with in situ tumors, and have highlighted the oncological safety of the preservation of the nipple-areola complex for patients with a negative intraoperative retroareolar frozen section. Many studies 1 3 9 evaluating the outcomes of patients who underwent NSM were performed with heterogeneous samples, including patients with invasive and intraepithelial tumors, and focusing on NSM with immediate breast reconstruction using prosthetic implants (saline-filled implants or tissue expanders) and autologous tissue flaps. 10 11 12 13 The recovery of breast reconstruction following mastectomy is a concern for surgeons. Kim et al. 14 showed that NSM with immediate breast reconstruction with implants is a safe and feasible procedure associated with good cosmetic results. In 2017, most of the NSM performed in Italy were reconstructed using prothesis and direct-to-implant reconstruction was gaining acceptance. 15 The aim of the present study was to retrospectively evaluate the oncological safety, complications and survival rates of patients diagnosed with pure DCIS who underwent NSM with immediate breast reconstruction with prostheses.

## Methods

The present retrospective study was performed according to ethical guidelines, and received approval from the Ethics in Research Committee of Hospital São Lucas and Hospital Albert Einstein. Patients with complete medical records were included, and all of them had beene operated on by the main author of the present study. Informed consent was waived by the institutional review board because of the retrospective nature of the study.

Between January 2004 and December 2018, 345 NSMs with immediate breast reconstruction were performed, 77 for in situ tumors. We excluded 5 patients who underwent reconstruction with tissue expanders, 4 patients with recurrence after BCS for invasive cancer, and 2 patients due to loss to follow-up (less than 3 months of follow-up). Patients who underwent risk-reduction NSM with an accidental finding of DCIS were included in the study. The tumor-to-nipple distance and tumor size were not exclusion criteria. Sentinel lymph node biopsy (SLNB) was performed for all patients in the affected breast. All patients were operated on by the same surgeon. The data was retrospectively evaluated through the medical charts, and the follow-up of the patients was updated during the appointments.

We analyzed 67 patients diagnosed with pure DCIS who underwent NSM with immediate implant-based reconstruction. The indications for NSM were risk-reduction breast surgery with an accidental finding of DCIS ( *n* = 4; 6%), multifocal disease ( *n* = 16; 23.9%), compromised margins after BCS ( *n* = 11; 16.4%), tumors ≥ 40 mm ( *n* = 16; 23.9%), and unfavorable relationships between tumor size and breast size or patient preference ( *n* = 20; 29.8%). The postoperative complications were defined as hematoma requiring drainage, infection, prolonged seroma formation, skin necrosis, partial nipple necrosis, total nipple necrosis, and prothesis extrusion.

The patients were followed by means of clinical examinations every six to twelve months for the first five years, followed by yearly exams thereafter. Imaging exams, such as ultrasonography, magnetic resonance, or mammography, were required based on patient complaints and after a physical examination.

The recurrences were diagnosed in clinical examinations or imaging exams, and all of the breast and axillary recurrences were biopsied and sent to pathology to confirm the diagnosis. Invasive or in situ local recurrence was defined as recurrence in the same breast and/or ipsilateral axilla.

All procedures were performed under general anesthesia using a periareolar, vertical, or inframammary incision. The NSM skin incision was chosen in accordance with breast type and method of reconstruction, and the majority of them were in the inframammary fold. The glandular tissue was removed respecting the plane of the subcutaneous fascia, carefully resecting breast tissue but leaving a sufficient amount of fat tissue to preserve blood flow and avoid flap necrosis. It is important to highlight that flap thickness varies among patients, since it is based on the amount of subcutaneous fat present in the breast.

An intraoperative histopathological examination of frozen sections of the retroareolar tissue was performed to confirm the absence of DCIS in the retroareolar margin, and all the patients presented tumor-negative margins. No cutoffs for margin status were used, and the postoperative histopathological examination confirmed that all samples were tumor-free.

**Fig. 1:**
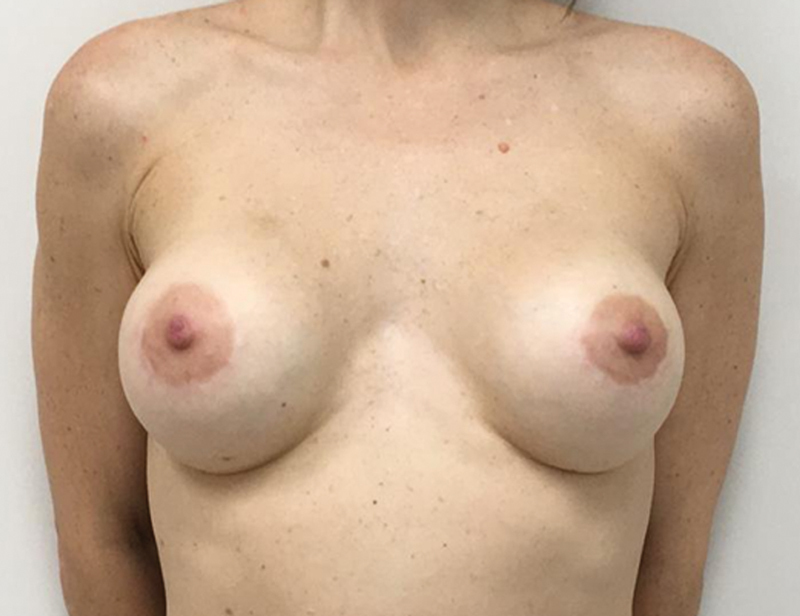
Preoperative bilateral NSM.

**Fig. 2:**
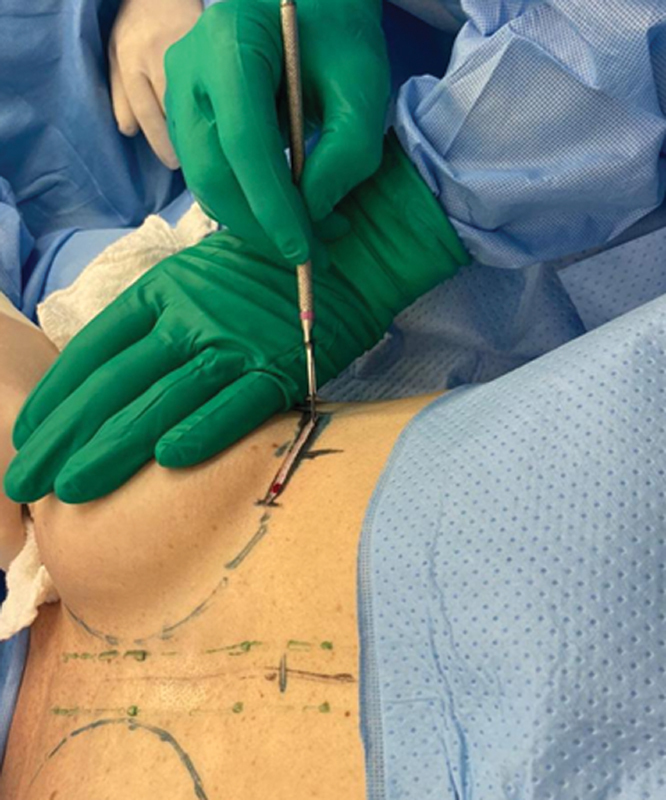
Inframammary skin incision.

**Fig. 3:**
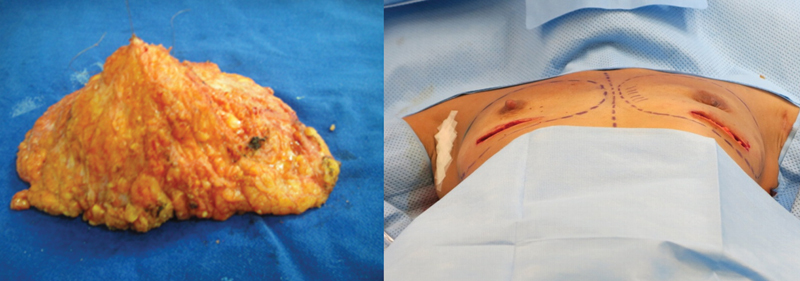
All breast tissue removed.

**Fig. 4:**
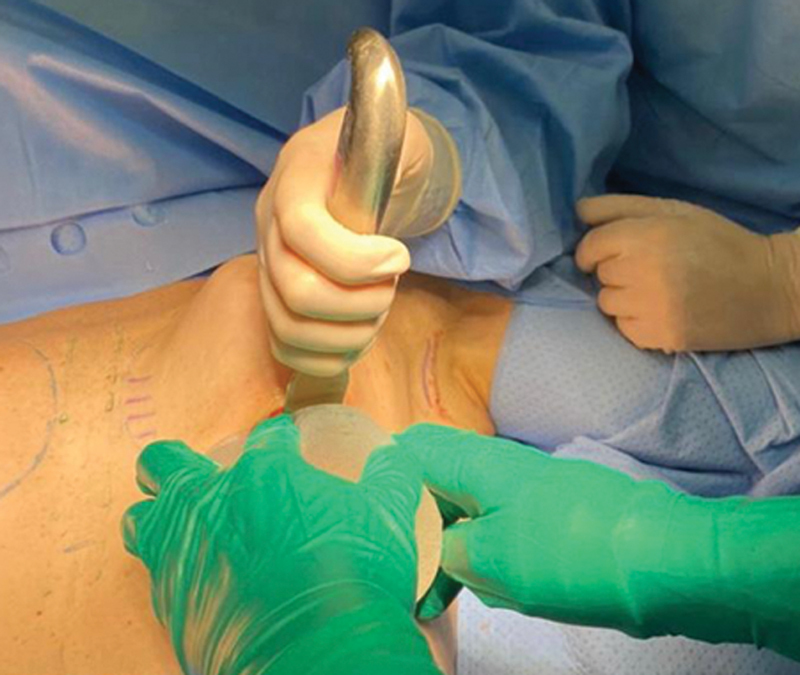
Inclusion of silicone implant.

**Fig. 5:**
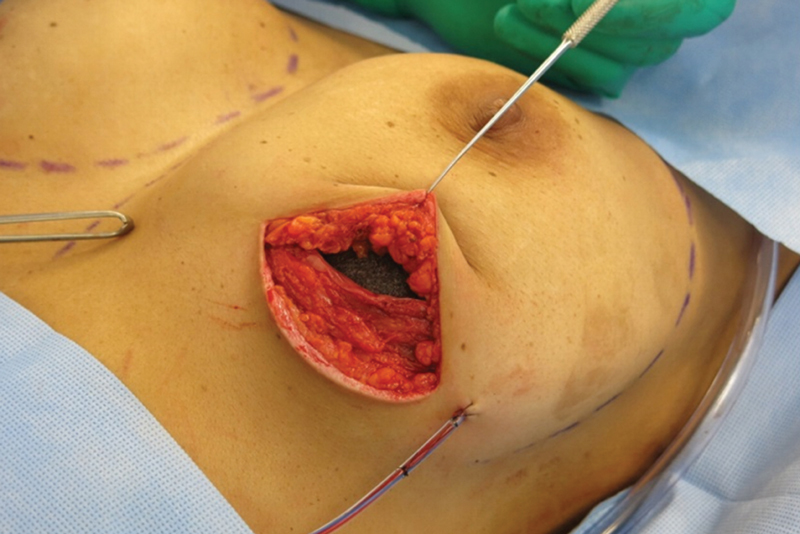
Breast reconstructed with silicone implant.

**Fig. 6:**
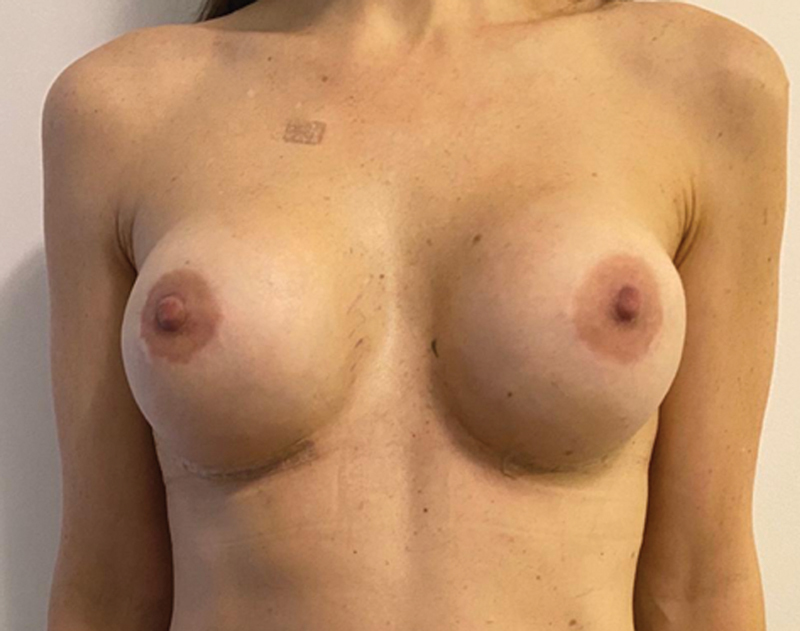
Postoperative bilateral NSM with immediate breast reconstruction with permanent silicone implants.

**Fig. 7:**
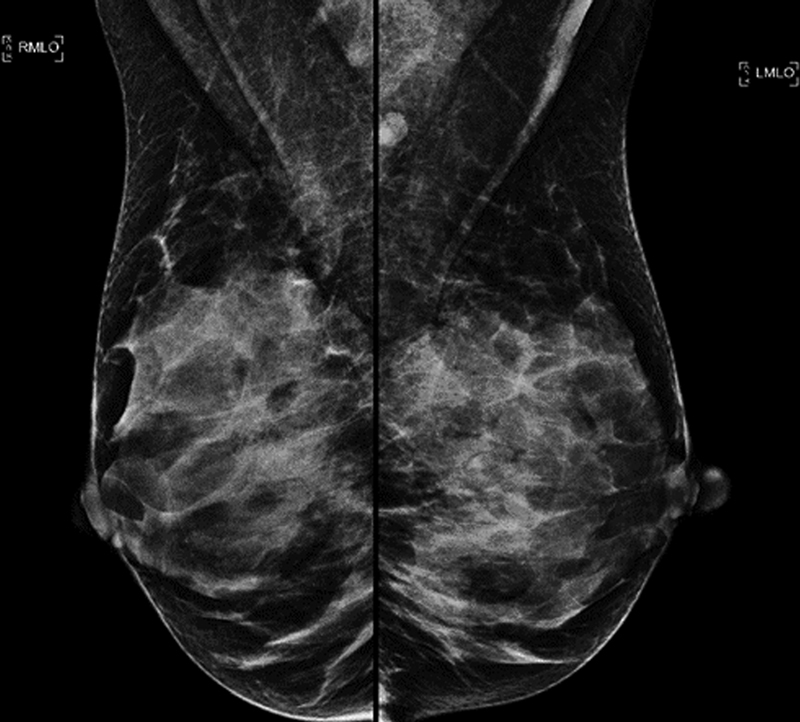
Preoperative imaging exam.

**Fig. 8:**
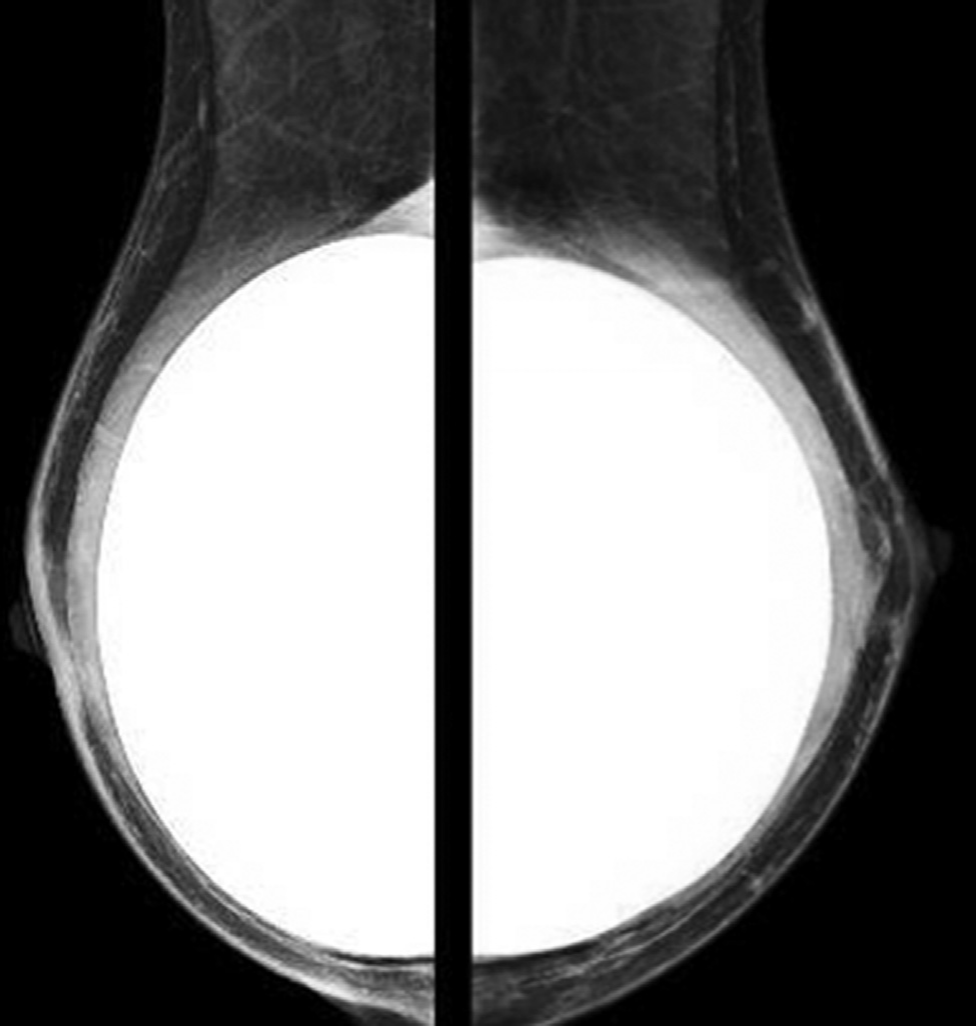
Postoperative imaging exam of the reconstructed breast with permanent silicone implants.

Patients with DCIS were submitted to SLNB, but not in the breast that underwent contralateral mastectomy. Immediate breast reconstruction was performed using subpectoral definitive prosthetic implants in every patient ( Figs. 1 , 2 , 3 , 4 , 5 , 6 , 7 , 8 ). None of these patients required the use of acellular dermal matrix implants.

The statistical analysis was performed with information from 67 patients. Descriptive statistics was used to summarize the characteristics of the patients. The quantitative variables were expressed as means and ranges, while the categorical variables were expressed as absolute and relative frequencies. Disease-free survival (DFS) was summarized using the Kaplan-Meier method and displayed graphically. The significance level for statistical differences was set at 0.05. The statistical analysis was performed using the SAS (SAS Institute, Inc., Cary, NC, US) statistical software, version 9.4.

## Results

We analyzed 67 patients who were diagnosed with pure DCIS and underwent 127 NSMs with implant-based immediate reconstruction between 2004 and 2018. The mean age of the patients was 46.8 years (range 30–75 years). The clinicopathological characteristics and treatment are listed in Table 1 .

**Table 1:** Patient, tumor, and treatment characteristics ( *n* = 67)

Characteristics	n	%
Age, years		
< 35	4	5.9
35–49	42	62.7
≥ 49	21	31.4
Menopause status		
Premenopausal	48	71.6
Postmenopausal	19	28.4
Previous cancer		
No previous history of breast cancer	56	83.6
Compromised margin after previous surgery	11	16.4
Genetic test		
Yes	18	26.8
Positive for mutations		
* BRCA1*	1	5.5
* BRCA2*	4	22.3
* P53 4*	1	5.5
* ATM*	1	5.5
* VUS ATM e P53*	2	11.2
Negative	9	50
No	49	73.2
Tumor size (mm)		
< 40	49	73.2
≥ 40	16	23.9
Unknown	2	2.9
Type of lesion		
Unifocal	36	53.7
Multifocal	31	46.3
Tumor grade		
1	7	10.4
2	26	38.9
3	32	47.8
Unknown	2	2.9
Systemic treatments		
Hormone therapy		
Yes	14	20.9
No	51	76.2
Unknown	2	2.9
Radiotherapy		
Yes	11	16.4
No	56	83.6
Reconstruction		
Implant	67	100

Abbreviations: ATM, ataxia-telangiectasia mutated; BRCA, breast cancer gene; P53, tumor protein p53.

Bilateral procedures were performed in 60 (89.5%) patients, and 7 (10.5%) surgeries were unilateral. In total, 2 (3.4%) patients underwent bilateral surgery due to the diagnosis of DCIS in both breasts, 10 (16.7%) patients presented a diagnosis of atypical hyperplasia in the contralateral breast, 7 (11.7%) patients were mutation carriers with high risk of developing breast cancer, and 41 (68.2%) patients underwent bilateral NSM by choice, looking for better symmetry and a better esthetic result. All patients underwent SLNB in the affected breast, and the lymph node was free of metastasis in every case. Unifocal lesions were found in 36 (53.7%) patients, and multifocal tumors were found in 31 (46.3%) cases. Most of the tumors were high-grade; 38.9% were grade-2, and 47.8%, grade-3. Only 10.4% of the tumors were grade-1 and data was missing from from 2 (2.9%) tumors. A total of 36 (53.7%) patients presented estrogen-receptor-positive (ER + ) tumors, 17 (25.4%), ER-negative (ER-) tumors, and data was missing from 14 (20.9%) tumors. Out of the 36 patients with ER+ tumors, 14 (38.9%) underwent hormone therapy (HT), and for the others patients the treatment was not indicated based on the risk/benefit of the hormonal treatment.

Immediate breast reconstruction was performed with silicone-filled implants for all patients. Frozen sections of the areolar flap's undersurface was performed for every patient, and the samples were tumor-free in the final analysis.

In the 69 procedures performed for DCIS, 3 (4.3%) complications occurred, including 2 (2.9%) cases of hematomas that required drainage, and 1 (1.4%) partial nipple necrosis. Full-thickness necrosis was defined as necrosis in the entire dermis requiring surgical intervention, such as debridement, delayed repair, and skin grafting, and partial nipple necrosis was defined as injuries that heal with conservative wound care, since they do not extend to the entire thickness of the dermis.

Adjuvant hormone therapy was administered to 14 patients (20.9%), and RT was administered to 12 patients (18%): because of the large extent of the superficial distribution of the DCIS, even with free margins. For one patient, the RT data were missing, and one patient chose not to undergo it. None of the patients submitted to RT presented complications. The patient with partial nipple necrosis presented type-1 diabetes.

During the mean follow-up of 60 months (range: 3 to 183 months), 6 (8.9%) patients presented local recurrence, and 2 (2.95%) cases occurred in the nipple-areola complex. From the 6 relapses, 4 were DCIS and 2 were invasive ductal carcinoma (IDC). None of the patients who presented local recurrence underwent RT, and only 1 patient was older than 50 years old.

**Fig. 9:**
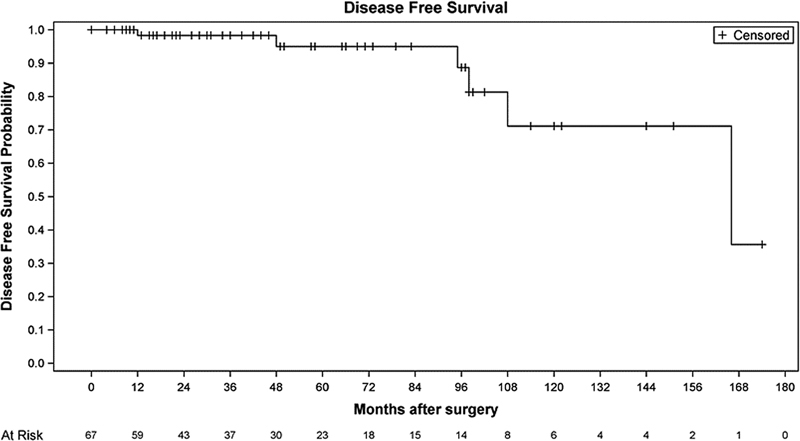
Disease-free survival ( *n* = 67).

Details regarding local recurrence are shown in Table 2 and Table 3 . None of the patients presented metastasis. The DFS rate was of 90% ( Fig. 9 ), and all patients were alive at the end of the 60-month follow-up.

**Table 2:** Local recurrence rates

Local recurrence	n	%
Same quadrant	2	2.9
Same breast in another quadrant	2	2.9
Nipple-areola complex	2	2.9

**Table 3:** Characteristics of the local recurrence

Previous cancer	Recurrence (months)	Age (years)	Surgery/ pathology	Lymph node status	Other treatments	Relapse	Survival
No	109	52	Bilateral NSM; 40 mm; multifocal; grade 3	SLNB LFN-	TMX	Nipple DCIS	Alive
No	12	47	Bilateral NSM; 17 mm; multifocal; grade 2	SLNB LFN-	None	Nipple DCIS	Alive
No	96	40	Unilateral NSM; 50 mm; unifocal; grade 2	SLNB LFN-	None	Same quadrant DCIS	Alive
No	48	38	Bilateral NSM; 45 mm; multifocal; grade 2	SLNB LFN-	None	Same quadrant DCIS	Alive
No	167	42	Unilateral NSM; 20 mm; multifocal; grade 3	SLNB LFN-	TMX	Other quadrant in same breast IDC	Alive
No	98	47	Bilateral NSM; 20 mm; multifocal; grade 3	SLNB LFN-	TMX	Other quadrant in same breast IDC	Alive

Abbreviations: DCIS, ductal carcinoma in situ; IDC, invasive ductal carcinoma; LFN, lymph node; NSM, nipple-sparing mastectomy; SLNB, sentinel lymph node biopsy; tMX, Tamoxifen.

## Discussion

Nipple-sparing mastectomy is a conservative approach for breast cancer with good rates of esthetic satisfaction from the patients. 16 17 The main concerns regarding the use of NSM are nipple necrosis and local and nipple recurrences. Different authors 1 3 9 11 18 19 have reported rates of local and nipple-areola-complex recurrence after NSM ranging from 0% to 11.6% and 0.7% to 4.8% respectively. The reported incidence of nipple necrosis after NSM ranges from 1.4% to 5.9%. 3 18 19 However, most of the published studies analyzed the rates of complications and recurrence after NSM in heterogeneous samples with invasive and noninvasive tumors. 1

The increasing number of NSMs has expanded the classic indications, and the number of NSMs for patients with DCIS has been increasing. Most of the studies 10 11 12 available in the literature regarding the rates of nipple complications and the oncological safety of NSM for patients diagnosed with pure DCIS used different techniques of breast reconstruction, such as prosthetic implants or an autologous tissue flap. Complications related to breast reconstruction after mastectomy are still a concern. The use of immediate implant-based reconstruction after NSM seems to be safe and feasible, and has been growing worldwide. 14 15 A retrospective study 20 with 435 patients who underwent NSM for invasive and in situ tumors with primary implant reconstruction reported a rate of 5.9% of skin flap ischemia/necrosis. In the present study, we pnly analyzed patients with immediate implant-based reconstruction, and we found a rate of 1.4% of nipple necrosis, which is lower than the rates in most of the previous reports of NSM for DCIS, including a large series 13 published in 2018 that reported a rate of nipple-areola-complex necrosis of 2.2% for in situ cancer. Our cumulative complication rates were also lower than those reported in previous studies, with only 4.3% of complications in 69 NSM procedures performed for DCIS. Leclère et al. 10 reported a rate of 17% of nipple-areola-complex necrosis and 5.3% of local recurrence in the long-term follow-up of 41 patients diagnosed with DCIS who underwent NSM. Despite the high rate of nipple-areola-complex necrosis, the locoregional recurrence rate for DCIS was low; however, the loss of patients over the mean follow-up period of 7.1 years (only 46% completed the follow-up) and the subsequent small sample size were limitations of the study. 10 In 2018, Lago et al. 11 evaluated the oncological safety of NSM for DCIS in 69 patients with a 10-year follow-up. The authors reported a rate of local relapse of 11.6%, and a low rate of nipple-areola-complex recurrence (1.4%). They did not observe cases of nipple necrosis after surgery either. 11 The current study evidenced a rate of locoregional recurrence (LRR) of 4.5%, and a rate of nipple-areola-complex recurrence of 3% in the 10-year follow-up. The authors demonstrated that characteristics such as negative progesterone receptor status, and tumor size ≥ 4 cm were related to an increased risk of developing LRR. Interestingly, margin status presented no statistical significance associated with LRR. 12

At a mean follow-up of 60 months, our local recurrence rate of 8.9%, including the nipple-areola-complex recurrence rate of 2.95%, was higher when compared with that of previous studies ( Chart 1 ).

**Chart 1:** Outcomes of NSM patients in previous studies

Authors	Year	N	Nipple necrosis (%)	LR (%)	Nipple-areola-complex recurrence (%)	OS (%)	Follow-up (months)
Petit et al. (2012) 1	2002–2007	162 in situ cases	−	4.9	2.9	95.5	Median: 50
Shimo et al. (2016) 18	2000–2013	425 NSMs (413 patients)	1.4	5.8	2.3	96.8	Median: 46.8
Manning and Sacchini (2016) 9	2000–2013	728 NSMs (413 patients)	0	0	0	97.3	Median: 49
Orzalesi et al. (2016) 3	2009–2014	1,006 NSMs (913 patients)	4.8	2.9	0.7	99.3	Mean: 91.7
Headon et al. (2016) 19	1970–2015	12,358 NSMs (10,935 patients)	5.9	2.38	−	−	Mean: 38
Leclère et al. (2014) 10	2000–2010	41 DCIS NSM patients	17	5.3	−	−	Mean: 85
Lago et al. (2017) 11	1984–2016	69 DCIS NSM patients	0	11.6	1.4	98.6	Mean: 142.6
Wu et al. (2020) 12	2003–2015	199 pure DCIS NSM patients	−	4.5	3	98.5	Median: 97
Frasson et al. (2021; present study)	2004–2018	67 pure DCIS NSM patients	1.4	8.9	2.9	100	Mean: 60

Abbreviations: DCIS, ductal carcinoma in situ; LR, local recurrence; OS, overall survival; NSM, nipple-sparing mastectomy.

All of the relapses in the tumor bed and nipple areola complex were cases of DCIS, and the two recurrences in another quadrant in the same breast were IDCs. We consider all patients with no involvement of the skin or nipple areola complex candidates for NSM; therefore, the characteristics of our patients, such as young age, tumors larger than 40 mm, multifocal tumors, and no criteria for the distance between the lesion and the skin or the nipple areola complex (since all margins were free) might have influenced the increased rate of local relapse found in the present study. Wu et al. 12 reported that tumor size ≥ 4 cm was risk factor for LRR in the univariate analysis; however, in the multivariate analysis, the statistical significance was borderline ( *p* = 0.064). 12 We observed a tendency of tumor multifocality to be a risk factor for local recurrence ( *p* = 0.061); however, due to the small sample size of the present study, we found no statistical difference when analyzing correlations between local recurrence and the clinicopathological characteristics and treatments. No cases of distant metastasis were observed in our patients.

For patients who underwent NSM, the benefit of RT has not been confirmed yet. However, a Cochrane review 21 confirmed the benefit of RT for all patients diagnosed with DCIS and submitted to BCS. Therefore, we chose to apply RT in patients with larger tumors. None of the patients who relapsed underwent RT after the first surgery. Radiotherapy was performed after the recurrence, and only one patient presented a new recurrence after it. Radiotherapy after BCS is the gold standard treatment for cases of early breast cancer, including DCIS. We hypothesize a possible relationship between RT and a reduced risk of developing ipsilateral breast cancer recurrence after NSM for DCIS, but new studies are necessary to confirm this potential benefit. 21

As observed in the National Surgical Adjuvant Breast and Bowel Project (NSABP) B-17 and B-24 randomized clinical trials for DCIS, 22 young patients with DCIS have an increased risk of developing invasive ipsilateral breast tumor recurrence; in the present study, only 1 patient who prsented relapse was older than 50 years of age. In our population, there were 48 patients aged ≤ 50 years, and analyzing only this population, we found a local recurrence rate of 10.4%. Approximately 19% of the young patients underwent RT; however, none of the patients who presented relapse underwent RT. The use of RT for local control in these patients might be an option.

The use of hormone receptors (ER, progesterone receptor [PR], human epidermal growth factor receptor 2 [HER2]) as prognostic biomarkers is still controversial for DCIS. Some authors have observed an association between HER2 positive and ER- DCIS with increased risk of recurrence, whereas in other studies these associations have not observed. 23 24 25 26 Endocrine therapy is indicated after BCS for patients with ER+ DCIS aiming to reduce the risk of local relapse and contralateral breast recurrence. 22 In the sample of the present study, HT was not indicated for all patients with ER+ tumors: ∼ 40% were treated with the hormone treatment. All patients that relapsed presented ER+ tumors, and half of them underwent hormone treatment. We evaluated the risk/benefit of hormone treatment for each patient presenting ER+ DCIS, considering the side effects and the risk of recurrence based on tumor size, grade, and young age. Indeed, since of all these patients were submitted to NSM and most of them underwent bilateral procedures, we considered that the risk of local and contralateral recurrence was diminished, and did not indicate HT for all ER+ patients

In the present study, all patients were alive by the end of the follow-up, and this result is consistent with the NSM findings reported by Lago et al., 11 2018, and Wu et al., 12 2020. In the long-term follow-up, Lago et al. 11 and Wu et al. 12 indicated overall survival (OS) rates of 98.6% and 98.5% respectively in patients diagnosed with DCIS who underwent NSM. Despite the expanded NSM indications applied in the present study, such as patients with compromised margins after a previous surgery, large and high-grade tumors, and no cutoff for distance between the tumor and the nipple-areola complex, the relapses did not interfere with patient survival in 60 months of follow-up.

The present study has several limitation,s such as the retrospective design of the analysis, the small sample size, and the short follow-up. External validation is needed to confirm our results.

## Conclusion

At a mean follow-up of 60 months, we demonstrated low complication rates and good survival in 67 patients who underwent NSM for pure DCIS with immediate implant-based reconstruction. The local recurrence rate was high, and characteristics such as young age, multifocal tumors, and no criteria for the distance between the lesion and the skin or the nipple-areola complex might have influenced the relapses found in the present study. We observed that the multifocality of the tumor might be a risk factor for local relapse; however further studies are needed to confirm this correlation. The present study supports that expanding the indication for NSM with immediate implant-based breast reconstruction to treat patients diagnosed with pure DCIS is acceptable when BCS is not an option and the patient wishes to preserve the nipple-areola complex.
